# Bacteriophage Effectively Rescues Pneumonia Caused by Prevalent Multidrug-Resistant *Klebsiella pneumoniae* in the Early Stage

**DOI:** 10.1128/spectrum.02358-22

**Published:** 2022-09-27

**Authors:** Lin Gan, Hanyu Fu, Ziyan Tian, Jinghua Cui, Chao Yan, Guanhua Xue, Zheng Fan, Bing Du, Junxia Feng, Hanqing Zhao, Yanling Feng, Ziying Xu, Tongtong Fu, Xiaohu Cui, Rui Zhang, Shuheng Du, Shiyu Liu, Yao Zhou, Qun Zhang, Ling Cao, Jing Yuan

**Affiliations:** a Department of Bacteriology, Capital Institute of Pediatrics, Beijing, China; b Department of Pulmonology, The Affiliated Children’s Hospital, Capital Institute of Pediatrics, Beijing, China; University of Exeter

**Keywords:** bacteriophage, *Klebsiella pneumoniae*, multidrug resistant, pneumonia

## Abstract

Pneumonia caused by multidrug-resistant (MDR) Klebsiella pneumoniae of sequence types ST11 and ST383 have highlighted the necessity for new therapies against these prevalent pathogens. Bacteriophages (phages) may be used as alternatives or complements to antibiotics for treating MDR bacteria because they show potential efficacy in mouse models and even individual clinical cases, and they also cause fewer side effects, such as microbiota-imbalance-induced diseases. In the present study, we screened two phages, pKp11 and pKp383, that targeted ST11 and ST383 MDR K. pneumoniae isolates collected from patients with pneumonia, and they exhibited a broad host range, high lytic activity, and high environmental adaptability. Both phages pKp11 and pKp383 provided an effective treatment for the early stage of pneumonia in a murine infection model without promoting obvious side effects, and cocktails consisting of the two phages were more effective for reducing bacterial loads, inflammation, and pathogenic injuries. Our findings support the application of phages as new medications for refractory ST11 and ST383 K. pneumoniae infections and emphasize the potential of enhancing phage therapy modalities through phage screening. These data provided important resources for assessing and optimizing phage therapies for MDR ST11 and ST383 infection treatment. However, substantial amounts of further work are needed before phage therapy can be translated to human therapeutics.

**IMPORTANCE** K. pneumoniae is recognized as the most common pathogen of hospital- and community-acquired pneumonia across the world. The strains of ST11 and ST383 are frequently reported in patients with pneumonia. However, the efficacy of antibiotics toward K. pneumoniae is decreasing dramatically. As a new approach to combat MDR bacteria, phages have exhibited positive clinical effects and efficacy as synergetic or alternative strategies to antibiotics. Thus, we screened two phages that targeted ST11 and ST383 MDR K. pneumoniae, and they exhibited a broad host range, high lytic activity, and high environmental adaptability. Both phages provided an effective treatment for the early stage of pneumonia in mice, and cocktails consisting of the two phages were more effective in reducing bacterial loads, inflammation, and pathogenic injuries. Although these data suggest that phages are effective alternatives or complements to antibiotics, more research is needed before they can be translated into therapeutics for humans.

## INTRODUCTION

Klebsiella pneumoniae, a Gram-negative bacterium that belongs to the family *Enterobacteriaceae*, appears to be widely distributed in various environmental niches such as water, soil, feces, hospitals, communities, animals, and humans (1). K. pneumoniae can cause a series of clinical conditions, such as pneumonia, meningitis, endophthalmitis, pyogenic liver abscess, septicemia, urinary tract infection, and even nonalcoholic fatty liver disease (2, 3). It is recognized as the most common pathogen of hospital- and community-acquired pneumonia and one of the most common nosocomial pathogens in the world (4). According to the latest report by the China Antimicrobial Surveillance Network (CHINET), K. pneumoniae accounts for 14.12% of clinical isolates in China. Without appropriate treatment, the mortality rates associated with K. pneumoniae infections can be as high as 50% (5).

Within the pneumonia-associated K. pneumoniae sequence types (STs), isolates of partial STs, such as ST11, ST23, ST25, ST65, ST268, and ST383, are frequently reported (6–8). Our previous study showed that ST11 and ST383 were also prevalent STs of pneumonia-associated K. pneumoniae isolates (8). Another recent study revealed that the general carriage of antibiotic-resistant genes and virulence genes establishes the basis for the high prevalence rate of some subtypes, such as ST11 (9).

The antibiotic resistance of K. pneumoniae is a serious problem across the world. Under constant evolutionary and antibiotic pressure, the treatment efficacy of most available antibiotics toward K. pneumoniae is decreasing dramatically, and multidrug-resistant (MDR) or extremely drug-resistant K. pneumoniae are frequently reported worldwide. Extended-spectrum β-lactam-producing and carbapenem-resistant K. pneumoniae have been identified as critical public health threats and have been named “superbugs” by the World Health Organization (10). In a multicenter clinical study that collated data from 14 provinces in China, evidence showed most carbapenem-resistant *Enterobacteriaceae* cases were coinfected with K. pneumoniae (491/664, 73.9%) (11). However, new antibiotics or combination therapies based on existing antibiotics have not exhibited satisfactory outcomes thus far. In addition, antibiotic-induced microbiota imbalance may lead to unexpected and unknown side effects because of the important role the microbiota plays in various diseases, such as metabolic and immune-mediated diseases (12, 13).

As a new approach to combat MDR bacteria, bacteriophage (phage) therapy has exhibited positive clinical effects and confers therapeutic benefits, with increasing evidences showing that phages are effective as synergetic or alternative strategies to antibiotics (14–16). In general, phages are abundant and highly strain-specific viruses that can precisely kill bacteria and metabolize themselves automatically after the host bacteria are removed *in vivo* (14). However, there are problems with phage application. Resistance to phages is frequently reported, and a previous study showed that phages can be seen as potential invaders and rapidly removed by the immune system (17). However, phage cocktails are considered promising approaches to overcome these problems (18).

To investigate the potential efficacy of phage therapies for prevalent MDR K. pneumoniae infections, we isolated two K. pneumoniae-specific phages from hospital sewages. We identified the biological and genomic characteristics, host ranges, and host bacterial receptors of the two phages. Furthermore, we established a prevalent MDR K. pneumoniae-caused acute pneumonia model to evaluate the therapeutic efficacy of a phage cocktail, as well as the individual phages. The findings will likely provide new perspectives on the benefits of phages to the clinical setting.

## RESULTS

### Phages pKp11 and pKp383 targeting MDR *K. pneumoniae* isolates.

A total of 194 clinical K. pneumoniae isolates previously collected from the Anhui, Beijing, Fujian, Henan, Jiangsu, Jiangxi, Shandong, Shanxi, and Zhejiang provinces in China were analyzed (8). The prevalent STs of these clinical isolates were ST23 (54/194, 27.84%), ST11 (39/194, 20.10%), ST700 (9/194, 4.64%), and ST383 (8/194, 4.12%), and the predominant subtypes of pneumonia-patient-sourced isolates were ST11 (38/70, 54.29%), and ST383 (8/70, 11.43%), which carried 4 to 23 (median, 4) antimicrobial resistance genes (8).

**FIG 1:**
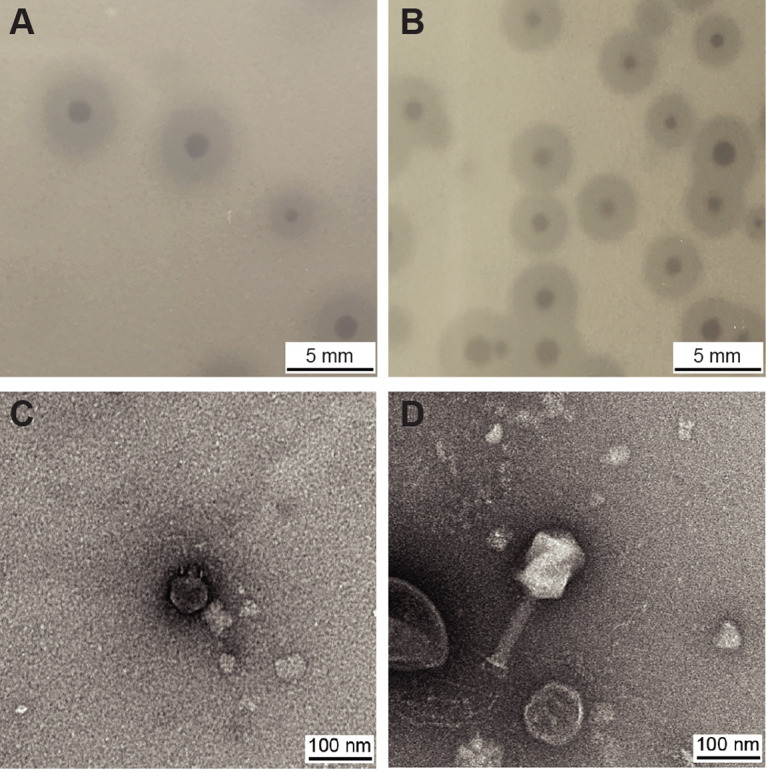
Morphologies of phages pKp11 and pKp383. (A and B) Phage plaques of pKp11 (A) and pKp383 (B) in a double-agar plate. (C and D) Phage morphologies of pKp11 (C) and pKp383 (D), as observed by TEM.

Two lytic phages were isolated from hospital sewage using prevalent K. pneumoniae isolates as the host strains. The phage that targeted an isolate of subtype ST11 (K. pneumoniae C10, JAJOTR000000000, collected from a pneumonia patient) was named pKp11 (GenBank accession number ON809559, CGMCC 45097), and the phage that targeted an isolate of subtype ST383 (K. pneumoniae C6, JAJOVD000000000, isolated from a pneumonia patient) was named pKp383 (GenBank accession number ON809560, CGMCC 45098). Phages pKp11 (Fig. 1A) and pKp383 (Fig. 1B) formed clear and round plaques with transparent centers on the agar plates. Transmission electron microscopy (TEM) showed that phage pKp11 was typical of the *Podoviridae* family, possessing an isometric head with a diameter of ~60 nm and a short tail (Fig. 1C), and phage pKp383 belongs to the *Siphoviridae* family and possessed an icosahedral head ~76 nm in diameter and a long noncontractile tail of ~126 nm (Fig. 1D).

**FIG 2:**
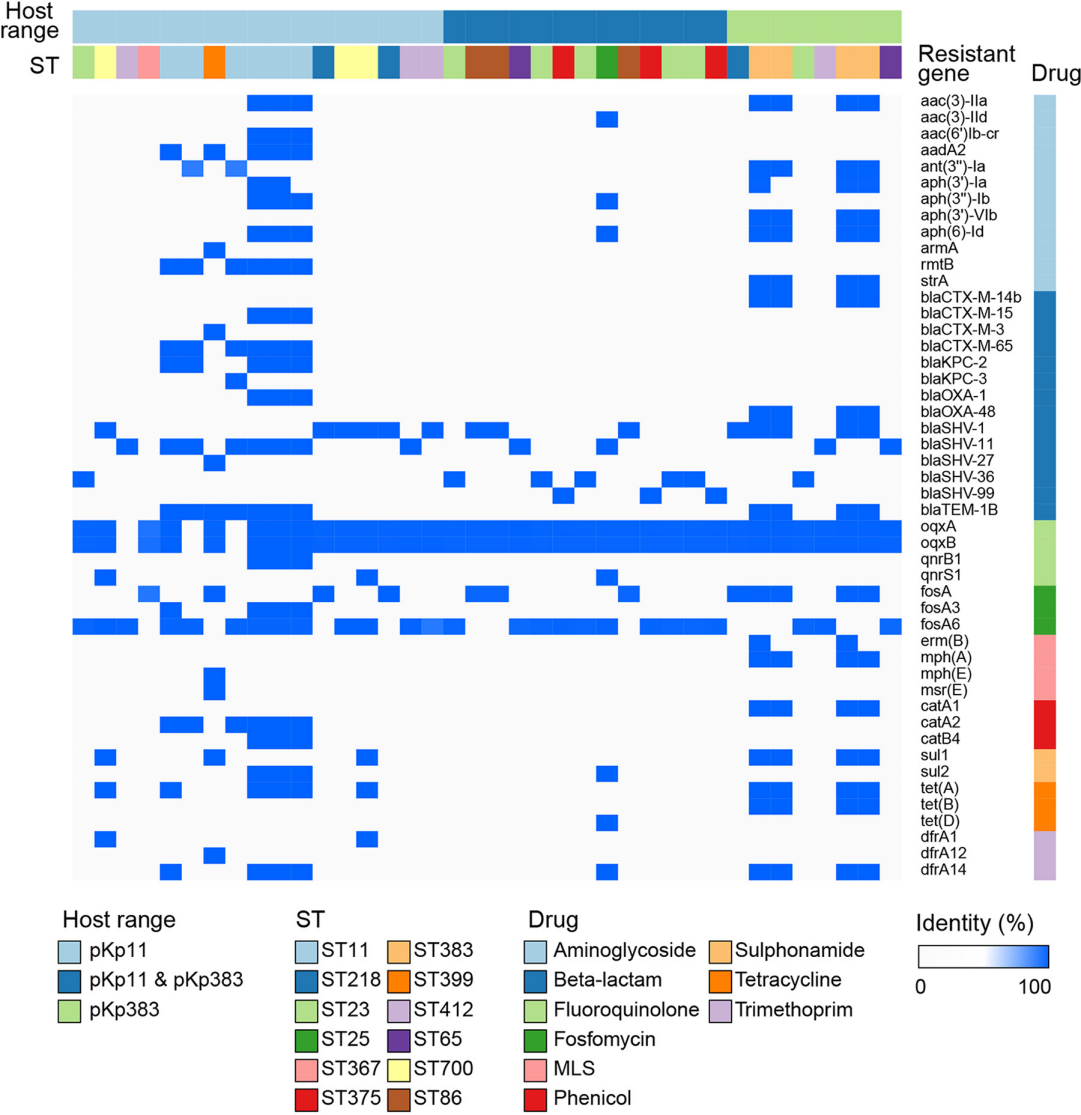
Heatmap of resistance genes in phages pKp11- and pKp383-targeted host K. pneumoniae isolates. Each row denotes a resistance gene, and each column denotes the resistance genes carried by an isolate. Squares above the isolates represent the phages’ host range and STs, and squares listed on the right side indicate the antibiotic-related resistance genes.

As shown in Fig. 2 and Table S1 in the supplemental material, phages pKp11 and pKp383 also lysed, in addition to K. pneumoniae C10 and C6, 30 (partial isolates of ST11, ST23, ST25, ST65, ST86, ST218, ST367, ST375, ST399, ST412, and ST700) and 21 (partial isolates of ST23, ST25, ST65, ST86, ST218, ST375, ST383, and ST412) K. pneumoniae isolates in 194 clinical isolates, respectively. All 51 host isolates carried multiple antimicrobial resistance genes, and host isolates of ST11 and ST383 carried significantly more (one-way analysis of variance [ANOVA], *P < *0.05) antimicrobial resistance genes than host isolates of other STs, which suggested a more urgent need to combat the resistance of ST11 and ST383 isolates in the clinic (Fig. 2).

### Life cycle parameters.

**FIG 3:**
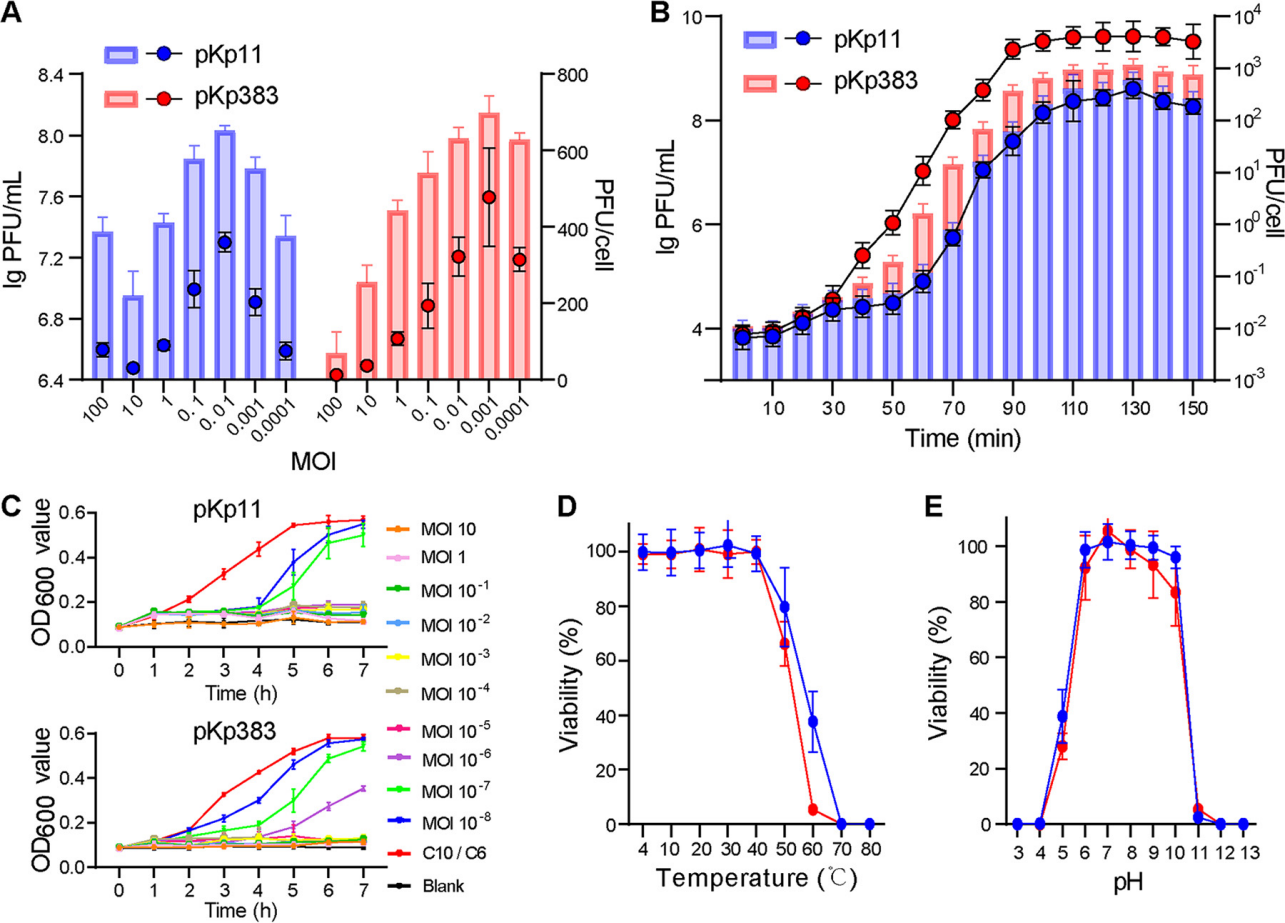
Life cycle parameters of phages pKp11 and pKp383. (A) MOIs of phages (*n* = 3). The final phage quantities are represented by columns and matched to the left *y* axis, the burst sizes are represented by dots and matched to the right *y* axis. (B) One-step growth curves of phages (*n* = 3). The final phage quantities are represented by dots and matched to the left *y* axis, the burst sizes are represented by columns and matched to the right *y* axis. (C) Kill curves of phages (*n* = 3). Host strains C10 and C6 were used as positive controls. (D) Stabilities of phages under temperatures 4°C to 80°C (*n* = 5). (E) Stabilities of phages under pH 3 to 13 (*n* = 5) at 37°C. Values are expressed as means ± the SD.

The optimal multiplicities of infection (MOIs) were 0.01 and 0.001 for phages pKp11 and pKp383, respectively (Fig. 3A). Subsequent experiments to determine the one-step growth curve and lytic ability against host strains were conducted under the optimal MOI. As shown in Fig. 3B, the latent period of pKp11 and pKp383 was 10 min, and the burst times and average burst sizes were 100 and 110 min and 262 and 501 PFU/cell, respectively. At an MOI of 10 to 10^−6^, pKp11 completely inhibited the host strain *K. pneumoniae* C10, and at an MOI of 10 to 10^−5^, pKp383 suppressed the host strain *K. pneumoniae* C6 (Fig. 3C). Phages pKp11 and pKp383 displayed good lytic ability against their host strains. In addition, pKp11 and pKp383 were stable between 4 to 50°C (Fig. 3D) and between pH 6 and 10 (Fig. 3E). Thermal and pH stability facilitate phage storage and transport. Taken together, these results indicate that pKp11 and pKp383 are potential candidates for the treatment of pneumonia caused by MDR K. pneumoniae strains.

### Genomic features.

**FIG 4:**
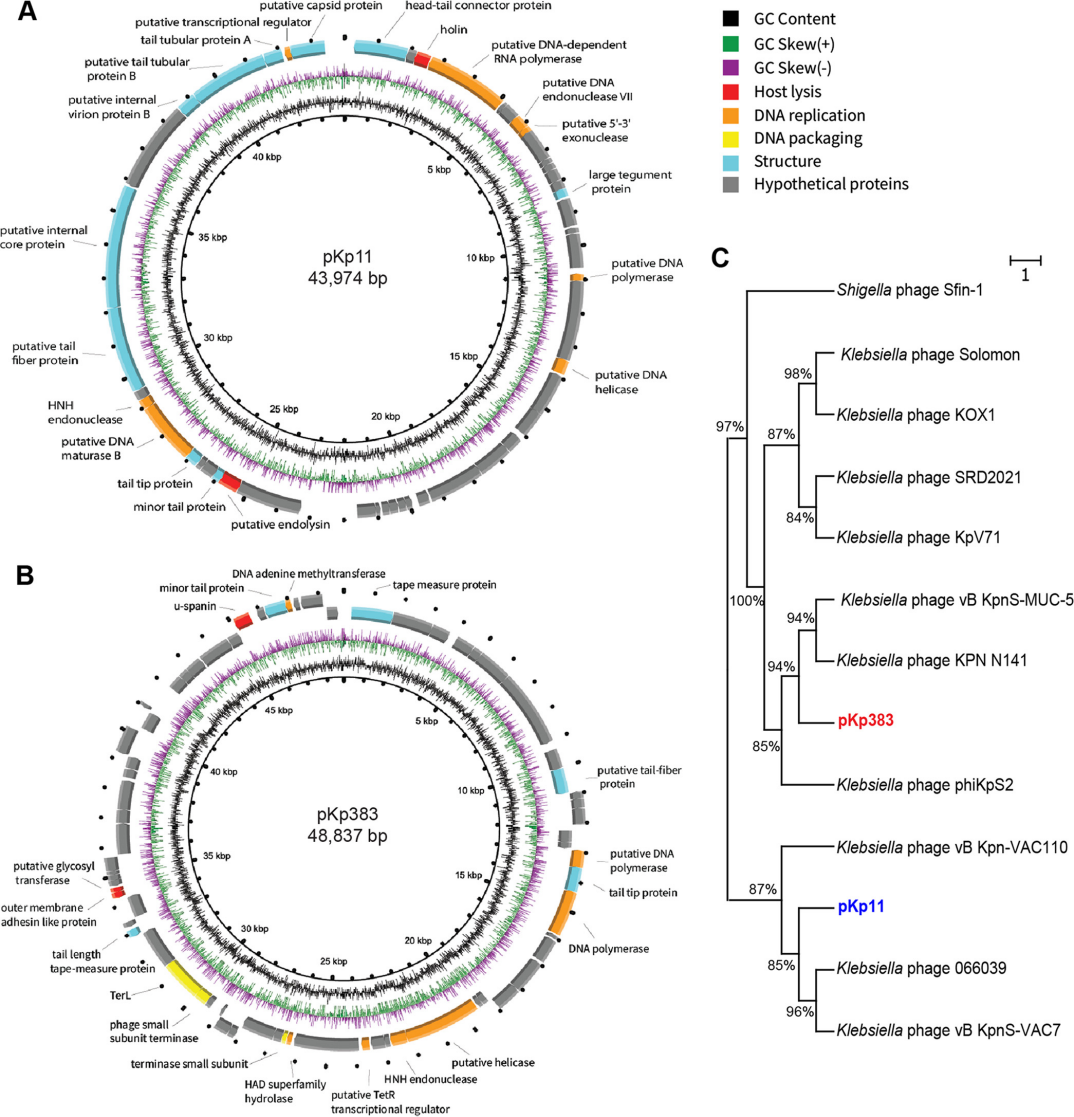
Genomic information for phages pKp11 and pKp383. (A and B) Genomic maps of phages pKp11 (A) and pKp383 (B). (C) Phylogenetic tree constructed from complete genomes using the maximum-likelihood method.

The genome of phage pKp11 was 43,974 bp in length with a 53.88% GC content, and coding domain sequence (CDS) prediction showed that pKp11 included 49 CDSs (see Table S2). Phage pKp383 was 48,837 bp in length with 70 open reading frames, and the GC content was 48.37% (see Table S3). As shown in Fig. 4A and B and Table S2, the protein profiles, including DNA replication proteins, DNA-packaging proteins, and structural proteins, were identified for phages pKp11 and pKp383, and they both carried no virulence, pathogenicity, or drug-resistance-related genes, indicating their potential applicability as therapeutic agents. The complete genome sequences of the phages were used to generate a phylogenetic tree. Phage pKp11 was clustered into the family *Podoviridae*, and pKp383 was grouped with *Siphoviridae*, findings that are consistent with the morphological results (Fig. 4C).

### Host receptors.

**FIG 5:**
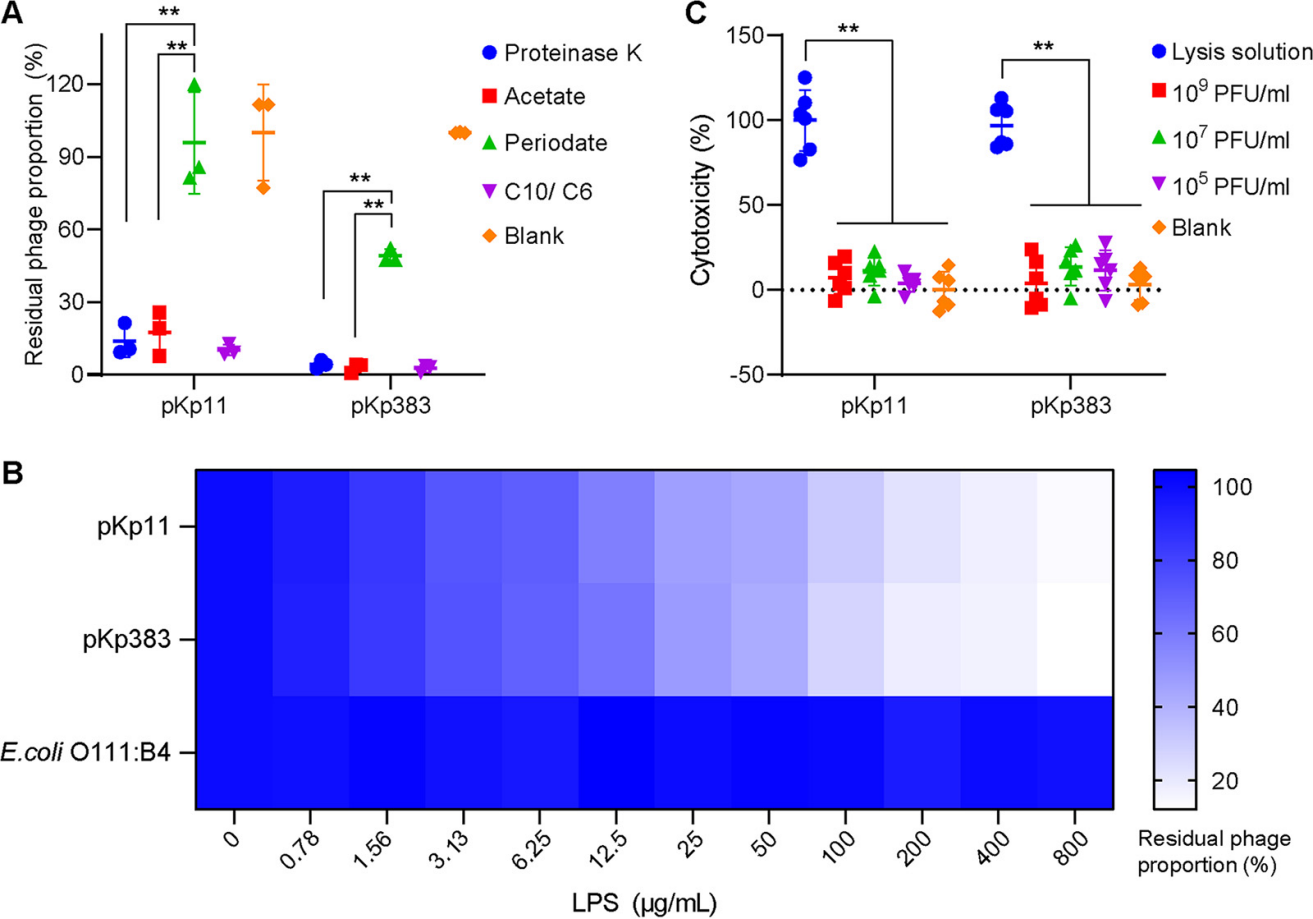
Host receptor identification and cytotoxicity of phages pKp11 and pKp383. (A) Effects of proteinase K and periodate on the adsorption of phages by K. pneumoniae isolates (*n* = 3). (B) Inactivation of phages by LPS derived from K. pneumoniae isolates (*n* = 3). The residual phage proportion is calculated as the percentage of phage quantities after proteinase K, periodate, or LPS compared to initial phage quantities. (C) Cytotoxicity of phages on A549 cells (*n* = 6). The cytotoxicity of phages on A549 cells after 3 h of incubation was assayed by measuring lactate dehydrogenase (LDH) levels in the culture supernatant. The group treated with lysis solution (0.8% Triton X-100) was used as a positive control. The cytotoxicity was calculated by using the following formula: cytotoxicity = (experimental LDH release − spontaneous LDH release)/(maximum LDH release − spontaneous LDH release). Values are expressed as means ± the SD. *, *P* < 0.05; **, *P* < 0.01 (one-way ANOVA).

Specific binding to the host strain receptor is the first step in phage infection, and outer membrane proteins or lipopolysaccharides (LPS) generally function as phage receptors on K. pneumoniae. In this study, outer-membrane-protein-targeting proteinase K and LPS-targeting periodate were used to screen the host receptors. As shown in Fig. 5A, the adsorption of phages pKp11 and pKp383 was not affected by proteinase K (one-way ANOVA, *P > *0.05), while phage-specific adhesion was significantly inhibited by periodate (one-way ANOVA, *P < *0.01) compared to the control group acetate. To further test the LPS receptors of pKp11 and pKp383, we measured their adsorption ability in medium containing 0 to 800 μg/mL LPS extracted from C10 and C6, and a group treated with LPS extracted from E. coli O111:B4 was used as a negative control. Approximately 12.5 μg/mL LPS inhibited half of the 2.5 × 10^9^ PFU of pKp11 or pKp383, and the activities of the phages showed direct correlations with LPS concentration, indicating that the LPS of K. pneumoniae is the specific receptor of pKp11 and pKp383 (Fig. 5B).

### Cytotoxicity of phages.

Since phage preparations for K. pneumoniae are usually treated through nasogastric administration, human lung A549 cell line was used for evaluating the cytotoxicity of the phages. The A549 cells were coincubated with different doses of the phages pKp11 and pKp383. As shown in Fig. 5C, the cytotoxicity of three doses of phage pKp11 was −10.82 to 22.71% and that of phage pKp383 was −6.48 to 27.77%. High (10^9^ PFU/mL), medium (10^7^ PFU/mL), and low (10^5^ PFU/mL) concentrations of phages all exhibited significantly less cytotoxicity than the control group (one-way ANOVA, *P < *0.01), which was treated with lysis solution (Promega, USA). No obvious differences were found between the phage-treated groups and the blank group (one-way ANOVA, *P > *0.05).

### Phage therapy in mouse models.

The efficiency and safety of phage therapy were examined *in vivo* using C57BL/6J mouse models of nasogastric infection. The minimum lethal dose (MLD) that triggered 100% death within 7 days was determined, and the MLDs of C10, C6, and SY1 (ST23, which was lysed by both pKp11 and pKp383) was 5 × 10^10^ CFU for all three strains. Thus, the pneumonia mouse models were intranasally infected with 2 × MLD (10^11^ CFU) of each K. pneumoniae isolate, and the dose used for phage therapy was 10^9^ PFU.

**FIG 6:**
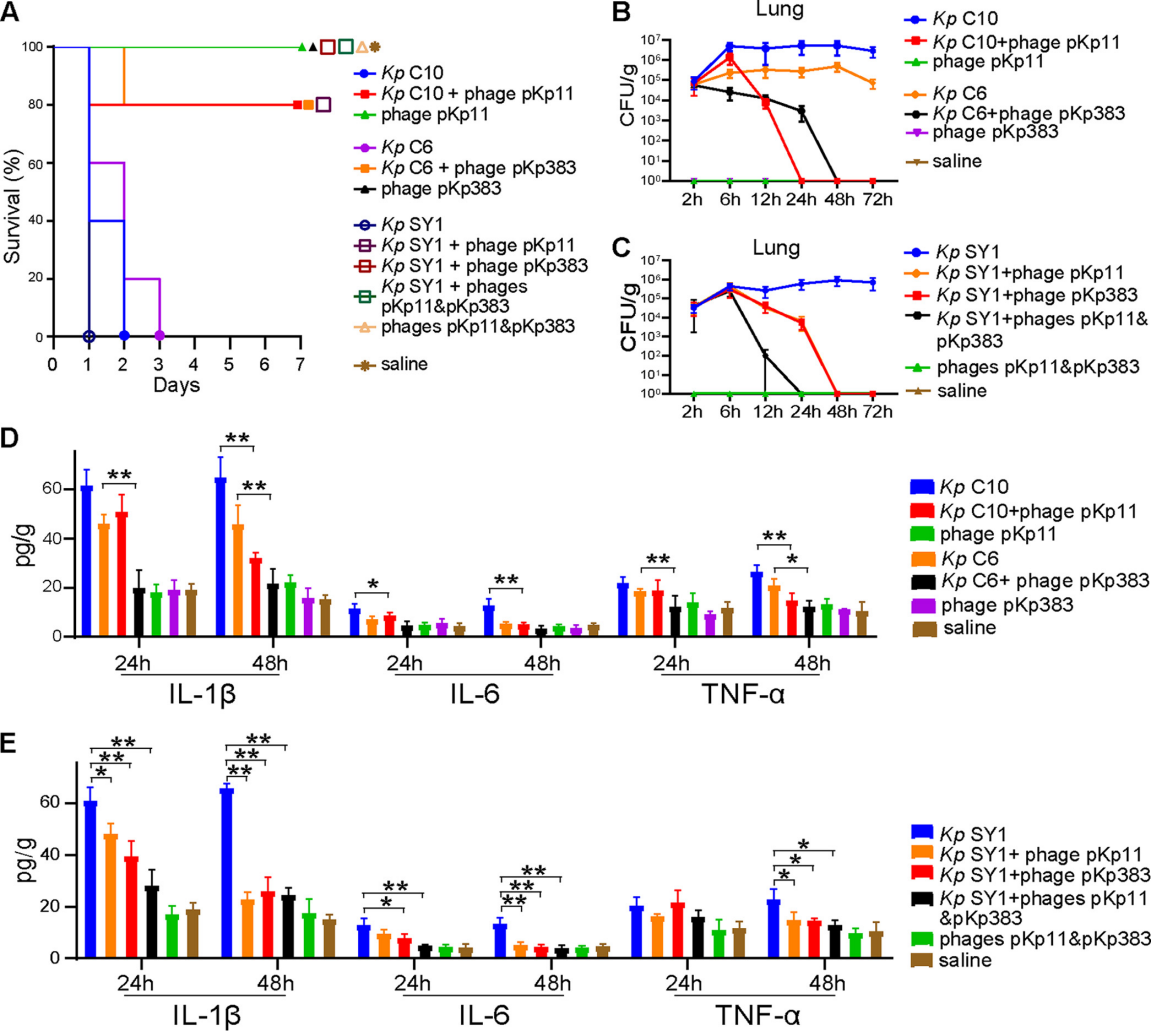
Phage therapy in mouse models. (A) Survival of mice infected with K. pneumoniae followed by phage treatment. (B and C) Changes in bacterial loads in the lungs were recorded for 72 h. (D and E) Proinflammatory cytokine levels in the lungs were measured at 24 and 48 h after K. pneumoniae infection. Values are expressed as means ± the SD (*n* = 5 mice/group). *, *P* < 0.05; **, *P* < 0.01 (one-way ANOVA).

All mice infected with K. pneumoniae isolates progressed quickly to severe infectious disease, while mice in the phage-treated group survived significantly better (log rank test, *P < *0.01) than those in the K. pneumoniae isolate-infected group (Fig. 6A). In C10/C6-infected groups, the survival rates were 100 and 80% for mice treated with pKp11 and pKp383, respectively. In groups infected with SY1, the survival rates were was 80% for mice treated with pKp11 and 100% for mice treated with pKp383 and cocktails (phages pKp11 and pKp383).

To further study the effects of phage on mouse models, mice were also sacrificed to evaluate the bacterial load, concentrations of inflammatory cytokines, and pathological injuries after the establishment of the pneumonia models. Bacterial loads in the lung were measured at 2, 6, 12, 24, 48, and 72 h postinfection. Both phages—pKp11 and pKp383—inhibited host strains in the lungs at 24 and 48 h (Fig. 6B). Compared to single-phage preparations, a phage cocktail of pKp11 and pKp383 exhibited a higher efficiency at suppressing host strains in the lungs (Fig. 6C). Since the host strains were eliminated in the lung at 24 or 48 h postinfection, we analyzed the parameters of samples at 24 or 48 h postinfection in subsequent experiments. To further evaluate the efficiency of the phages in treating pneumonia, we examined the concentrations of cytokines, including interleukin-1β (IL-1β), IL-6, and tumor necrosis factor alpha (TNF-α), and pathological injuries at 24 and 48 h postinfection. In the single-phage-treated groups, both pKp11 and pKp383 decreased the amounts of inflammatory cytokines by 48 h (one-way ANOVA, *P < *0.01 or 0.05) but not by 24 h (Fig. 6D and E). Although the IL-6 levels were not significantly decreased by phage pKp383 over 48 h, the concentration of IL-6 still showed a downward trend. Notably, the phage cocktails rapidly inhibited inflammation within 24 h (Fig. 6E).

**FIG 7:**
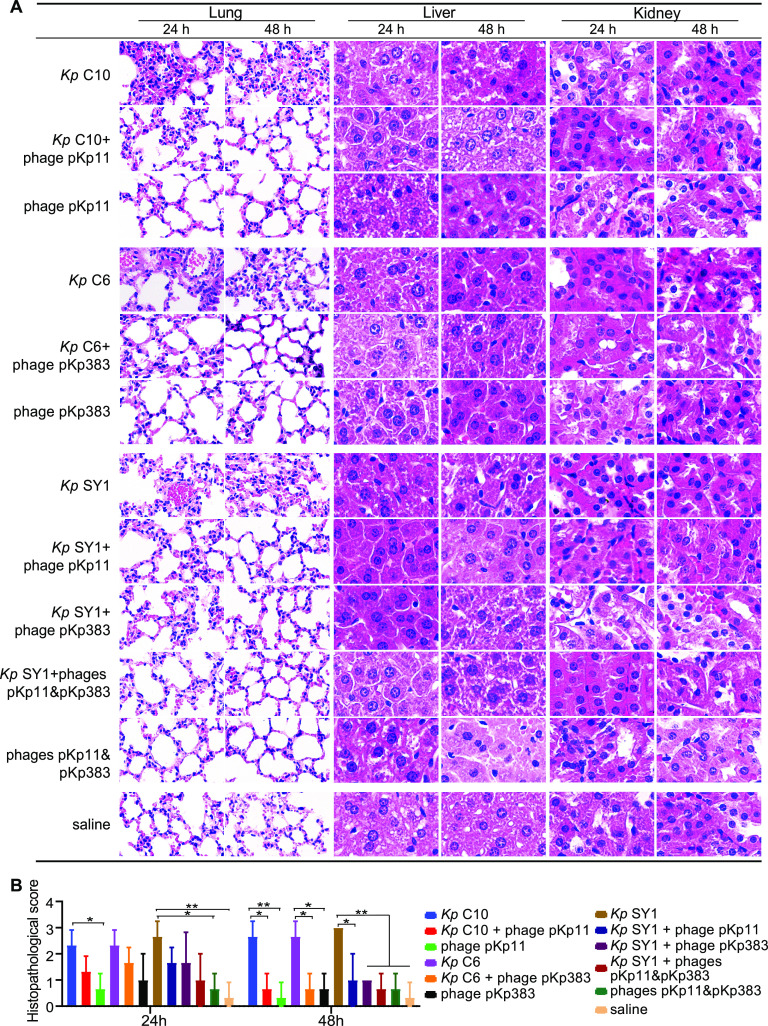
Histopathological analysis of mouse models. (A) Histopathological analysis of the lungs, liver, and kidneys, which were observed at 24 and 48 h after K. pneumoniae infection and captured under an optical microscope (200×). The tissues were stained with hematoxylin-eosin. The nucleic acids, including components such as the nucleus, were stained dark blue, and the proteins, including components such as cytoplasm, were stained pink. (B) Histopathological scores of lung tissue sections. The histopathological score was related to the following distribution: 1, thickened alveolar walls; 2, edema; and 3, tissue parenchymatous lesions such as congestion and hemorrhage. Tissue sections were evaluated by a trained pathologist with the following scores: 0, no pathological lesion; 1, mild; 2, moderate; and 3, severe.

After K. pneumoniae infection, pathological injuries, such as lung tissue structure destruction, alveolar wall thickening and fusion, pulmonary interstitial edema, hemorrhage, and inflammatory cell infiltration, were observed at 24 and 48 h postinfection *in vivo* (Fig. 7A), and the histopathological scores were evaluated with scores 0 to 3 (Fig. 7B). Consistent with the bacterial loads and changes in cytokines, mice treated with phage cocktail at the early stage of pneumonia showed less lung tissue damage within 24 h than mice treated with single-phage preparations (Fig. 7). In addition, no histopathology changes to the liver or kidney were observed in any phage-treated group (Fig. 7A), suggesting that the phages may be used as a medication for the treatment of K. pneumoniae-infected patients without severe side effects.

## DISCUSSION

MDR K. pneumoniae are widely distributed worldwide and can cause pneumonia or other invasive diseases in humans. K. pneumoniae isolates of ST11 and ST383 have spread across the globe, and most of these isolates carry resistance and virulence genes (7, 19, 20). Our previous study also showed that ST11 and ST383 are the predominant subtypes in China (8). However, current antibiotic therapies are not specific for K. pneumoniae and can cause side effects because they remove both pathogenic and beneficial commensal bacteria (21). Phages have been proposed as potential interventions to combat infections caused by MDR K. pneumoniae strains (22). Although experimental phage therapy cases are frequently reported, data that can be used to guide and justify clinical phage usage remain limited. In this study, we screened two phages that targeted MDR K. pneumoniae isolates and demonstrated that phage therapy, especially phage cocktails, effectively rescued mice with prevalent K. pneumoniae ST11 and ST383 isolate-caused pneumonia in the early stage.

Phage screening is vital for ascertaining the clinical effects of phage therapy, which is the point to culminating positive outcomes. Phages pKp11 and pKp383, which were isolated using prevalent pneumonia-sourced isolates as host strains, lysed partial isolates of ST11 and ST383, which carry multiple drug resistance genes, and exhibited broad host range, high lytic activity, and high environmental adaptability. Large burst sizes and short latent periods which were shown for phages pKp11 and pKp383, were verified to contribute to phages’ ability to efficiently lyse host bacterial cells (23). These two highly efficient lytic phages can adequately inhibit bacteria growth *in vitro*. Human body temperatures can reach a maximum of 40°C, while phages could experience up to 50°C during storage and transportation (24). Both phages can maintain high activity from 37°C to 50°C. In addition, both phages—pKp11 and pKp383—recognized LPS as receptors for adsorption, accounting for their specificity to the host strains of ST11 and ST383. LPS plays an important role in the pathogenic process of K. pneumoniae infections (25). Phage-resistant K. pneumoniae strains which recognized phage with LPS have been reported in recent years. However, most isolates with mutant LPS would be attenuated or avirulent and pose no serious problem for human health or phage therapy (26, 27). The phages investigated in this study not only have indispensable properties for phage therapy but also carry no virulence or resistance genes, suggesting that pKp11 and pKp383 have potential as agents for treating pneumonia.

From the perspective of their application, phages pKp11 and pKp383 lysed 51 MDR K. pneumoniae isolates, including isolates of ST11 and ST383, which are the prevalent subtypes in China and around the world, indicating their great application potential in curing pneumonia. Previous studies demonstrated that phage has the ability to improve survival and reduce antibiotic resistance *in vivo* (28, 29). Both phages pKp11 and pKp383 rapidly attenuated the bacterial loads and cytokine concentration and were associated with reduced tissue damage in the lungs, indicating that phage therapy is very effective in the pneumonia model. Previous studies showed that phages targeting host strains regulate proinflammatory and anti-inflammatory cytokines in mice and impact symbiotic bacteria and metabolomic profiles by cascade effects on interbacterial interactions (30–32). Collectively, our data also showed that phage therapy downregulated the expression of the proinflammatory cytokines IL-1β, IL-6, and TNF-α in the lungs, reducing the risk of a cytokine storm and the resulting poor clinical outcomes, which suggests that phage therapy could regulate proinflammatory effects beyond a reduction in bacterial load and pathogenic injury. In addition, no proinflammatory cytokine increase was found in groups treated with a single-phage preparation or phage cocktails, thus demonstrating the safety of phage therapy. Further study of anti-inflammatory cytokines, symbiotic bacteria, and metabolic profiles is needed to provide more practical significance and application value. Notably, a higher efficacy was observed in the experimental mice treated with phage cocktails. Consistent with this result, another study also verified that phage cocktails achieved better clinical outcomes than single-phage preparations *in vivo* (18). Phage cocktails broaden the host bacterial range, minimize the development of phage-resistant mutants, reduce infection severity, and prevent phage elimination by the immune system, thereby promoting the long-term effectiveness of the therapy (18, 23, 33). Further studies on phage cocktails and the evolution of phage resistance are needed before phages can be applied to treating K. pneumoniae-caused pneumonia.

Overall, our data demonstrated that phage therapy is effective for treating ST11 and ST383 infections in the early stage and induces no obvious side effects in mouse models. Although these data suggest that phages are effective alternatives or complements to antibiotics, substantial additional research is still needed before these phages can be translated into therapeutics for humans. A deeper understanding of phage-bacterium interactions and phage-resistant mutants *in vivo* will be vital for maximizing the efficacy of phage therapies in the future.

## MATERIALS AND METHODS

### Bacterial strains, cells, medium, and culture conditions.

K. pneumoniae isolates C10, C6, and SY1 were collected from pneumonia patients and incubated in Luria-Bertani (LB) medium (Oxoid, Ltd., UK) at 37°C. Human lung carcinoma cells A549 were cultured in Dulbecco modified Eagle medium containing 10% fetal calf serum (Gibco, USA) at 37°C with 5% CO_2_.

### Antimicrobial resistance genes analysis of bacterial host strains.

An *in silico* profile of antibiotic resistance genes was predicted by software Resfinder (version 4.0); the Resfinder database includes resistance genes related to aminoglycosides, beta-lactams, colistins, fluoroquinolones, fosfomycins, fusidic acid, glycopeptides, macrolides, lincosamides, and streptogramin B (MLS), nitroimidazoles, oxazolidinones, phenicols, pseudomonic acid, rifampicin, sulfonamides, tetracyclines, and trimethoprim (34). All resistance genes are listed on the Center for Genomic Epidemiology website (https://cge.cbs.dtu.dk/services/ResFinder/database.php).

### Isolation and purification of phages.

Phages were isolated from untreated hospital sewage using K. pneumoniae isolates C10 and C6 as host strains. The hospital sewage was centrifuged at 10,000 rpm for 5 min to remove large particles, and the supernatant was filtered through a 0.22-μm syringe filter to eliminate bacteria. Then, 200-μL filtrates and 100-μL log-phase samples of host strains were introduced into 5 mL of LB broth, followed by shaking culture for 2 h. The mixture of 100-μL clear cultures and 50-μL host strains (log-phase) were then incubated on a double-layer of agar overnight. The resulting single-phage plaques were purified three times using a double-layer plaque assay.

### Host range identification.

The host ranges of the phages were examined against 194 clinical K. pneumoniae isolates collected from nine provinces of China using a standard spot test (8, 35). Approximately 10^6^ PFU of phages in 10 μL of LB broth were spotted onto a lawn of host strains and incubated overnight. The strains sensitive to the phages formed a clear and transparent plaque on the plate.

### Transmission electron microscopy.

Phage particles were negatively stained with 2% (wt/vol) uranium acetate (pH 7.0) and observed by TEM operated at 80 kV.

### One-step growth curve.

The optimal MOI was identified by using a double-layer plaque assay. Phages and log-phase host strains were introduced into LB broth at MOIs of 0.0001, 0.001, 0.01, 0.1, 1, 10, and 100, respectively. After shaking culture for 2 h, the optimal MOI-treated group showed the highest phage quantities on a double-layer plate. For the one-step growth curve, the host strain was infected with each phage at the optimal MOI, and the phage quantities were calculated every 10 min over 150 min. The burst sizes of the phages were calculated from the proportion of the final number (150 min) of phages to the initial number (0 min) of bacteria (*n* = 3) in a one-step growth curve assay (36).

### Lysing ability of host strains.

The lytic activity of the phages toward the host strains was tested at an MOI of 10^−8^ to 10 with 10-fold dilutions. Then, the optical densites at 600 nm were tested on a microplate reader once an hour for each group.

### Thermal and pH stability.

Approximately 10^8^ PFU phages were incubated in LB medium at 4°C to 80°C and pH 1 to 14 (changed medium pH by 1 M HCl and 1 M NaOH) for 1 h, respectively. Next, the phage quantities were identified by using a double-layer plaque assay.

### Phage receptor determination.

To test whether proteinase K or periodate affects phage adsorption, K. pneumoniae isolates were pretreated with 20 μg/mL proteinase K (Solarbio, China), 100 mM periodate (Sigma, USA), and 50 mM sodium acetate (Sigma) for 3 h at 37°C and then coincubated with phage at room temperature for 5 min. The residual phage proportion was calculated as the percentage of phage quantities after proteinase K, periodate, or lipopolysaccharides treatment compared to the initial phage quantities. To further validate the phage inactivation caused by LPS, the LPS were extracted from host strains using an LPS extraction kit (catalog no. 17141; Intron Biotechnology, South Korea) and used in a phage inactivation test as previously described (37). Phages treated with LPS of Escherichia coli O111:B4 (Sigma) were used as a control group.

### Genome sequencing and analysis.

The genomic DNA of phages was extracted using the phenol-chloroform method, as previously described (38). Whole-genome sequencing of DNA was performed on an Illumina HiSeq 2500 platform (Berry Genomics Corp., China). Genome information was annotated by Prokka and PHASER (http://phaster.ca/) (39). A phylogenetic tree was constructed from the complete genomes using the maximum-likelihood method.

### Cytotoxicity assay.

A cytotoxicity assay was used to measure the lactate dehydrogenase (LDH) release that denotes the cell membrane damage, and the cytotoxicity of the phages was tested using an A549 cell model with an LDH kit (Promega) according to the manufacturer’s instructions. A549 cells (8 × 10^4^) were cultured in a 96-well plate and infected with high (10^9^ PFU/mL), medium (10^7^ PFU/mL), or low (10^5^ PFU/mL) concentrations of phages. After 3 h of incubation, the supernatant was collected for LDH determination (8). The cytotoxicity was calculated by using the following formula: cytoxicity = (experimental LDH release − spontaneous LDH release)/(maximum LDH release − spontaneous LDH release).

### *K. pneumoniae* infection and phage therapy in mouse models.

Seven-week-old male C57/6J mice (Charles River, China) and the experimental procedures involving all mouse models were approved by the medical ethics committee of the Capital Institute of Pediatrics. The procedures were carried out by a licensed individual (license number DWLL2021009). The MLD of K. pneumoniae isolates was evaluated in mouse models. As previously described, groups of 10 mice were infected intranasally with 10-fold dilutions of log-phase bacteria (5 × 10^7^, 5 × 10^8^, 5 × 10^9^, 5 × 10^10^, and 5 × 10^11^ CFU per mice) and monitored for 7 days (40, 41). For pneumonia mouse model establishment, groups of 5 mice were intranasally infected with 2× MLD of K. pneumoniae isolate and monitored for 72 h. Mice intranasally infected with saline were used as the control group, and 10^9^ PFU of phages were administered at 2 h postinfection to the phage-treated groups. Five mice were euthanized to monitor bacterial loads in the lungs at 2, 6, 12, 24, 48, and 72 h postinfection. The lung tissues were also used for cytokine (IL-1β, IL-6, and TNF-α) level determination, and the liver and kidney tissues were used for hematoxylin-eosin staining at 24 and 48 h postinfection. As previously described, the histopathological score of lung tissue sections was related to the following distribution: (i) thickened alveolar walls, (ii) edema, and (iii) tissue parenchymatous lesions, such as congestion and hemorrhage. Tissue sections were evaluated by a trained pathologist and assigned scores as follows: 0, no pathological lesion; 1, mild; 2, moderate; and 3, severe (42).

### Statistical analysis.

Data are presented as means ± the standard deviations (SD) and were analyzed using two-way ANOVA using SPSS 20.0 (IBM Corp., USA). Differences were judged statistically significant at a *P* value of <0.05 (*) or <0.01 (**).

### Data availability.

Whole-genome sequencing files of phages were submitted to National Center for Biotechnology Information (https://www.ncbi.nlm.nih.gov/nuccore/), and the accession numbers are ON809559 and ON809560, respectively. For specific genome accession numbers of bacteria, please see Table S1 in the supplemental data.
